# 282 Stitching Up Stewardship: Evaluation of Weekly Handshake Antimicrobial Stewardship Rounds with General Surgery

**DOI:** 10.1017/ash.2026.10643

**Published:** 2026-06-23

**Authors:** Laura Calderwood, Sara Mirza

**Affiliations:** 1 Cherokee Nation Operational Solutions and Division of Viral Diseases, CDC; 2 U.S. Centers for Disease Control and Prevention

## Abstract

**Background:** Outbreaks of norovirus and other enteric diseases are common in long-term care facilities (LTCFs) such as nursing homes and assisted living facilities. During the COVID-19 pandemic, LTCFs enacted strict infection control measures which, combined with societal non-pharmaceutical interventions, may have limited the introduction of other infectious diseases, including norovirus, into these settings and reduced disease transmission temporarily. However, a newly predominant strain of norovirus was associated with abnormally high outbreak activity in the 2024-25 winter season. We describe changes to trends in norovirus outbreaks in LTCFs reported to the National Outbreak Reporting System (NORS) and CaliciNet, a strain surveillance platform, during the last decade. **Methods:** State and territorial health departments voluntarily report foodborne, waterborne, and other enteric outbreaks to the Centers for Disease Control (CDC) and Prevention through NORS. Public health laboratories report laboratory-confirmed norovirus outbreaks and genotypes to CDC through CaliciNet. Merged data from both systems were analyzed across four time periods: pre-pandemic, August 2015 – March 2020; pandemic, April 2020 – July 2021; post-pandemic, August 2021 – July 2024; and the 2024-25 season, August 2024 – July 2025. Both laboratory-confirmed (positive specimens from ≥2 case-patients or as defined by the reporting site) and suspected (<2 positive specimens) etiology outbreaks were included. **Result:** In total, 16,196 enteric LTCF outbreaks were reported to NORS and/or CaliciNet from August 2015 – July 2025, of which 12,905 (80%) outbreaks with 342,712 associated cases were attributed to norovirus. The average number of outbreaks reported over 12 months was 1,505 (7,022 total) during the pre-pandemic period, 256 (341 total) during the pandemic period, and 1,085 (3,256 total) during the post-pandemic period. The predominant genotype during these periods, GII.4 Sydney[P16], caused 42% of outbreaks with genotype data available. During the 2024-25 season, 2,286 LTCF norovirus outbreaks were reported (52% higher than the pre-pandemic period) and the predominant genotype (76%) was GII.17[P17]. Outbreak size (median [IQR]: 21 cases [11–35]) and duration (median [IQR]: 8 days [5–14]) were consistent across periods. Hospitalization rates were lowest during the pandemic period (1.8%) and highest during the 2024-25 season (3.2%). **Conclusion:** The low burden of LTCF norovirus outbreaks during the COVID-19 pandemic highlights the impact of non-pharmaceutical interventions in preventing outbreaks in this setting. The increased norovirus activity in the 2024-25 season associated with a new predominant strain suggests that more proactive laboratory testing in response to outbreaks is needed in this at-risk population to monitor trends in norovirus

